# Plausible Role of Estrogens in Pathogenesis, Progression and Therapy of Lung Cancer

**DOI:** 10.3390/ijerph18020648

**Published:** 2021-01-14

**Authors:** Claudia Musial, Renata Zaucha, Alicja Kuban-Jankowska, Lucyna Konieczna, Mariusz Belka, Antonella Marino Gammazza, Tomasz Baczek, Francesco Cappello, Michal Wozniak, Magdalena Gorska-Ponikowska

**Affiliations:** 1 Department of Medical Chemistry, Medical University of Gdansk, 80-211 Gdansk, Poland; claudia.musial@gumed.edu.pl (C.M.); alicjakuban@gumed.edu.pl (A.K.-J.); mwozniak@gumed.edu.pl (M.W.); 2 Department of Clinical Oncology and Radiotherapy, Medical University of Gdansk, 80-214 Gdansk, Poland; rzaucha@gumed.edu.pl; 3 Department of Pharmaceutical Chemistry, Medical University of Gdansk, 80-416 Gdansk, Poland; lucyna.konieczna@gumed.edu.pl (L.K.); mariusz.belka@gumed.edu.pl (M.B.); tomasz.baczek@gumed.edu.pl (T.B.); 4 Department of Biomedicine, Neurosciences and Advanced Diagnostics (BiND), University of Palermo, 90127 Palermo, Italy; antonella.marino@hotmail.it (A.M.G.); francesco.cappello@unipa.it (F.C.)

**Keywords:** estrogens, lung cancer, sex hormones, lung adenocarcinoma, estrogen receptor, 17β-estradiol, *p53*, A549, non-small cell lung cancer, NSCLC

## Abstract

Malignant neoplasms are among the most common diseases and are responsible for the majority of deaths in the developed world. In contrast to men, available data show a clear upward trend in the incidence of lung cancer in women, making it almost as prevalent as breast cancer. Women might be more susceptible to the carcinogenic effect of tobacco smoke than men. Furthermore, available data indicate a much more frequent mutation of the tumor suppressor gene-*p53* in non-small cell lung cancer (NSCLC) female patients compared to males. Another important factor, however, might lie in the female sex hormones, whose mitogenic or carcinogenic effect is well known. Epidemiologic data show a correlation between hormone replacement therapy (HRT) or oral contraceptives (OCs), and increased mortality rates due to the increased incidence of malignant tumors, including lung cancer. Interestingly, two types of estrogen receptors have been detected in lung cancer cells: ERα and ERβ. The presence of ERα has been detected in tissues and non-small-cell lung carcinoma (NSCLC) cell lines. In contrast, overexpression of ERβ is a prognostic marker in NSCLC. Herein, we summarize the current knowledge on the role of estrogens in the etiopathogenesis of lung cancer, as well as biological, hormonal and genetic sex-related differences in this neoplasm.

## 1. Introduction

In the developed countries, lung cancer is the most frequent malignancy and is responsible for about 1 million deaths annually. The overall survival rate involving this tumor is about 10%. The decisive trigger is long-term smoking. Available data indicate that only 20% of lung cancer cases develop in non-smokers [1,2]. Other environmental factors include pollution, exhaust fumes, ionizing radiation, mycotoxins, second hand smoke, occupational exposure to chemicals such as chromium, nickel, asbestos, polycyclic aromatic hydrocarbons, arsenic, vinyl chloride and radioactive gas—radon [1,2,3,4,5]. Susceptibility to the disease is also genetically determined [6,7]. The WHO classification distinguishes two main types of lung cancer: small cell carcinoma (SCLC) and non-small cell carcinoma (NSCLC) [6]. The latter is divided into subtypes including squamous cell carcinoma and adenocarcinoma [6].

## 2. Lung Cancer—Short Review

Lung cancer is the leading cause of mortality in women and men worldwide, and is considered to be a major global epidemic [7]. 

**Figure 1:**
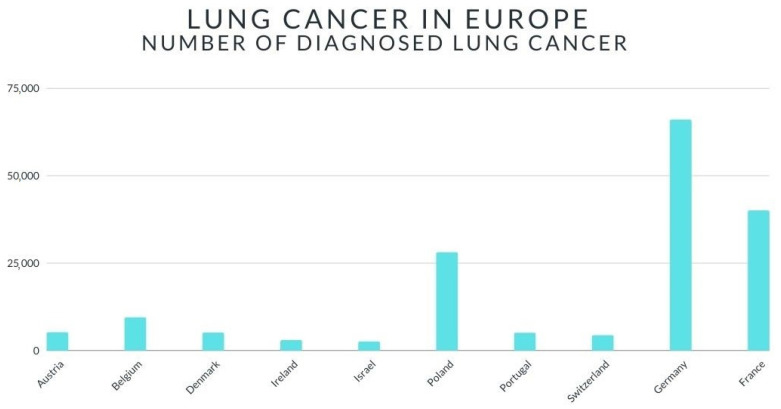
The graph presents number of persons with diagnosed lung cancer.

**Figure 2:**
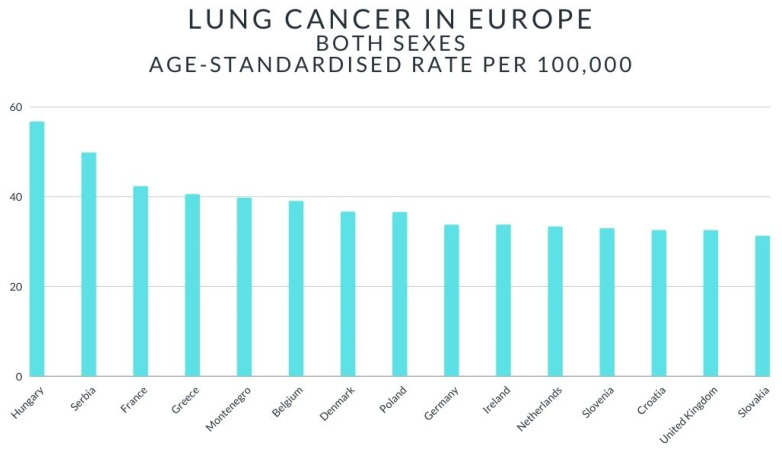
The graph shows a ranking of both sexes age-standardised rate per 100,000.

In Europe, lung cancer ranks second in terms of incidence. According to the data, as many as 23% of lung cancer cases worldwide occur in Europe [8]. In 2018, an estimated 1.8 million people developed lung cancer [8]. Figure 1 shows a graph showing the number of lung cancer cases (statistics for 2018) [8]. In Austria, lung cancer is the second most common cancer among men—2940 cases, and the third most common cancer among women—2202 cases [8]. In Belgium, 9400 people were diagnosed with lung cancer in 2018 [8]. Similarly to the Austrian population, lung cancer is the second most common cancer among Belgian men [8]—15% of all cases, and the third most common cancer among Belgian women—8% [8]. Moreover, it is the most common cause of cancer deaths among both women (15%) and men (30%). In France, lung cancer is the second most common cancer in both women and men [8]. In Germany, more than 66,000 cases of lung cancer were diagnosed in 2018-more than 27,000 women and over 39,600 men [8]. According to the data, in Poland, lung cancer has been diagnosed twice as often among men than among women [8]. In the United Kingdom, lung cancer is diagnosed in 1 in 17 women and 1 in 13 men [8]. Statistical analysis shows that in Sweden, lung cancer causes the highest mortality in both women and men [8]. The data presented in Figure 2 indicate that in 2018, Hungary had the highest rate of lung cancer [9].

The vast majority of lung cancer cases are caused by smoking tobacco, with smoking being a documented factor in the development of lung cancer [1,2,3]. Free radical oxygen is the main component of tobacco smoke. The substance may cause oxidation of guanine DNA nucleobases to form 8-oxoguanine (OGG1). Published data suggest that there is an increased risk of lung cancer in smokers with low OGG1 activity [8]. Tobacco smoke contains over 60 carcinogens, including aromatic hydrocarbons such as nitrosamines and benzopyrene, carbon monoxide (chad), tar, phenol, cresol, formaldehyde and hydrogen cyanide [10,11,12]. Polycyclic aromatic hydrocarbons activated by cytochrome P450 enzymes can bind to DNA [10,11,12]. Enzymes that catalyze the glutathione-S reaction protect against a DNA reaction that causes the formation of DNA adducts [10,11,12]. Chronic and too frequent adduct formation might cause gene mutations that could lead to the development of lung cancer. Nicotine inhibits apoptosis and promotes tumor cell growth in lung epithelial cells. Available studies indicate that non-smokers are more likely to survive than smokers, regardless of other prognostic factors. Quitting smoking has a direct impact on reducing lung cancer risk [13,14,15]. Exposure to tobacco smoke in the environment increases the risk of lung cancer by as much as 10% to 15% [15,16,17,18,19].

Other factors that contribute to the development of lung cancer include exposure to air pollution, chronic infections, asbestos, radon gas, viruses (JC virus, simian virus 40, BK virus, cytomegalovirus and human papillomavirus) and sex hormones [1,2,3].

Susceptibility to the disease is also genetically determined [11,12,13,14,15,16,17,18,19,20]. Gene mutations, which result in changes of protein expression, such as Bax, *p53* or Bcl-2 [21,22], have a significant impact on the prognosis of lung cancer patients [13]. The disease onset is very insidious without any early symptoms. In more advanced stages, the main symptoms at diagnosis include: chronic cough, chest pain, shortness of breath, recurrent pneumonia and hemoptysis [23,24]. Lung cancer spreads easily to the bones causing pathological fractures and bone pain, to the liver causing significant weight loss, jaundice, nausea and abdominal pain, to the adrenal glands—which is symptomless or leads to endocrine alterations—or to the brain causing seizures, paresis, balance disorders, sensory disturbances, convulsions and headache [23,24].

There are two main histological classes of neoplasms derived from respiratory epithelial cells: small cell lung cancer (SCLC) in 15% of cases and non-small cell lung cancer (NSCLC) further divided into adenocarcinoma, squamous cell carcinoma and large cell carcinoma in the other 85% of all cases [3,22,23,24,25]. The most aggressive is SCLC, which rapidly spreads to the regional lymph nodes and parenchymal organs vessels. Another characteristic includes extreme predilection to the brain and other parenchymal organs. One of the first cell lines to analyze molecular biology of lung cancer was established in the 1960s, while the SCLC cell line was established in the 1970s. Lung cancer treatment, depending on the type and stage of cancer, includes surgery, chemotherapy, targeted therapy and radiation therapy [25,26,27,28]. Cisplatin is the backbone of the most efficient chemotherapy regimens, usually combined with either gemcitabine, vinorelbine, etoposide or taxanes [29,30,31,32]. 

Nowadays, in locally advanced NSCLC, which is not amenable towards radical surgery, the best treatment results are obtained by using upfront concomitant chemoradiotherapy followed by a maintenance immune checkpoint inhibitor. The results of phase III prospective randomized clinical trial PACIFIC showed that durvalumab—an anti-PDL-1 monoclonal IgG1 kappa antibody—significantly prolonged overall survival, as compared with placebo (stratified hazard ratio for death, 0.68; 99.73% CI, 0.47 to 0.997; *p* = 0.0025) [33]. Interestingly, a meta-analysis evaluating the effectiveness of anti-PD1 inhibitors and standard chemotherapy in female patients did not show a clear benefit from immune checkpoint inhibitors. In contrast, male patients had a 24% reduction in the risk of progression [34]. These results raise the question about the gender-related mechanisms and the value of sex as an independent prognostic factor for anti-PD1 or anti-PD-L1 blockade. Female patients may have more potent immune systems or develop immune-resistant lung tumors [34]. 

Patients diagnosed with adenocarcinoma harboring druggable mutations are currently offered molecularly targeted medications directed against EGFR, ALK, ROS, Her2 new, MET, TRK or any other even less frequent alterations. In SCLC, the treatment progress is less evident than in NSCLC, but immunotherapy impacting the CTLA4 or PD signaling can be added to standard chemotherapy and combined with radiation [27,28,29]. 

Better treatment results in lung cancer female patients have been noticed in several clinical trials. Pinto et al. retrospectively reviewed available data to explore differences in gender outcomes in NSCLC. The meta-analysis showed a 27% reduction in the risk of death in female patients. In six trials evaluating EGFR TKI (tyrosine kinase inhibitors) in adenocarcinoma of the lung, there was a 10% reduction in the risk of progression in women compared to men (HR = 0.44 vs. 0.34 for men and women, respectively). However, it is essential to mention that important prognostic factors such as ethnicity or smoking status have not been included. Another meta-analysis by Pujol et al. did not show any differences in benefit from cetuximab (an anti-EGFR antibody) between genders in the subgroup analysis [35].

Regarding another biological compound—anti-angiogenic bevacizumab—the results of clinical trials are inconclusive. A meta-analysis by Soria et al. showed no correlation between gender and the treatment effect [36]. Female patients with lung adenocarcinoma harboring ALK that was rearranged and treated with ALK inhibitors such as crizotinib or ceritinib obtain similar benefits as men [37].

## 3. Sex Differences in Lung Cancer

In contrast to men, available epidemiological data indicate an upward trend in the incidence of lung cancer in women, despite a 50 percent reduction of women smokers [33,34,35,36,37]. The decisive role is attributed to female sex hormones, mainly estrogen, which is a steroid. The main hormone referred to as the reproductive hormone is 17-β-Estradiol-E2, which is synthesized in the ovaries under the influence of the luteinizing and follicular hormones [33,34,35,36,37]. 

There are two types of estrogen receptors (ER): ER alpha (ERα, also known as ESR1) and ER beta (ERβ, ESR2). Many studies have shown a correlation between hormone replacement therapy and the risk of cancer mortality in women [38,39,40]. The ERβ receptor has been demonstrated as essential in healthy lung tissues, where it is necessary to maintain the extracellular matrix. The ERβ estrogen receptor is characterized by genomic and non-genomic activity. The non-genomic effect concerns vasodilatation [41,42,43,44,45,46]. It becomes apparent 5 to 20 min after exposure to estrogen, and does not require changes in gene expression. In contrast, the genomic effect of estrogens involves protection against atherosclerosis and inhibition of the response to injury. In addition, high ERβ expression is associated with a poor prognosis of advanced NSCLC [41,46]. mRNA analyses were performed, comparing lung tumors with low and high levels of ERβ receptors. Tumors exhibiting high ERβ expression have been characterized as signalers via fibroblast growth factors, which are the autocrine signaling loop and contribute to the progression of lung cancer and pluripotency of human embryonic stem cells. Moreover, cancer stem cells (CSCs) are responsible for both primary tumor growth and metastasis. Wnt pathway, Notch pathway and Hh pathway routes are responsible for differentiation and pluripotency of CSCs [47]. In vitro studies indicate that in the lung, ER estrogen receptors may interact with the epidermal growth factor receptor (EGFR) during carcinogenesis. A clinical study of 180 women showed an increased risk of developing adenocarcinoma in patients receiving hormone replacement therapy (HRT) [48]. It was also noticed that women taking HRT for extended time periods are more susceptible to the harmful effects of tobacco smoke [39,40,41]. Genetic factors are another important determinant of developing lung cancer, which is independent of the smoking status [42,49]. Notably, available data indicate that it is the reason why women bear a generally higher risk than men [50,51]. One of the main well-studied factors is the role of CYP1A1 (cytochrome P450) gene expression [52]. The CYP1A1 gene codes for the phase I enzyme, which is involved in the metabolism of polycyclic aromatic hydrocarbons (PAH), contained in tobacco smoke as well as other types of smoke produced by burning different products. This mechanism prevents the pre-carcinogen from turning carcinogenic. Circulating female steroid hormones are believed to influence the modulation of PAH enzyme expression due to interaction with receptors in the patients’ lungs. In addition, it has been shown that the expression of steroid receptors is much more common in women than in men [52]. Available clinical studies indicate that overexpression of the CYP1A1 gene has a clear effect on the increased risk of lung cancer in women. It is also known that due to impaired DNA repair mechanisms, platinum-based chemotherapy has a better therapeutic effect. The antitumor activity of platinum drugs is mainly based on the mechanism of deformation of the DNA structure, thanks to the formation of stable DNA adducts [51,52,53,54]. 

## 4. Estrogens Short Review

**Figure 3:**
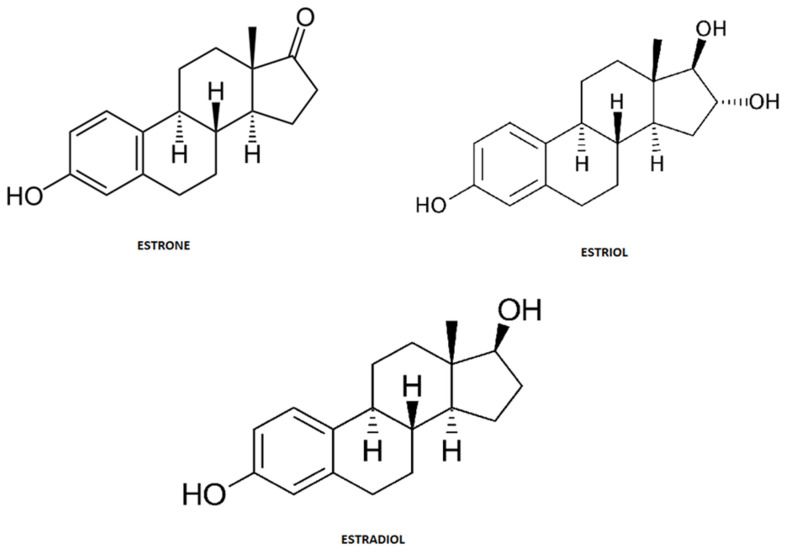
Chemical structure of steroid hormones.

Steroid hormones are endogenous estrogens that include estrone (E1), estriol (E3) and 17β-estradiol (E2) (Figure 3). These structural and biogenic hormones are derived from cholesterol C17. LDL-cholesterol is the major reactant necessary for the synthesis of steroid hormones, which is called steroidogenesis [52,53,54]. 

Cholesterol is metabolized in a number of enzymatic pathways [55,56]. The process of their creation depends on the aromatization of androgens [55,56]. In addition, they have the ability to bind to the protein receptor (ER) as well as to diffuse through the cell membrane. Direct penetration through the cell membrane into the cytosol occurs due to the properties of the lipophilic structure. In addition, estrogens are included in the group of pleiotropic hormones [55,56]. 

**Figure 4:**
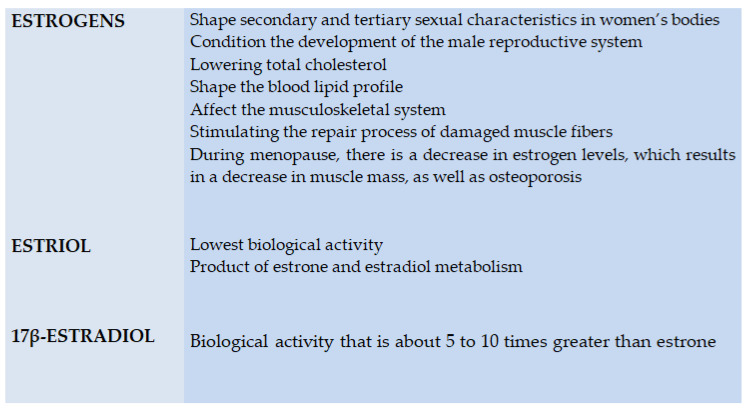
Biological activity of steroid hormones.

The two main estrogens also named “parent” estrogens—estrone, and estradiol—are low-molecular steroids of lipophilic nature acting as agonists of estrogen receptors ERα and ERβ [56]. However, estrogen-like activity should be also attributed (with varying extent) to a range of estrogen metabolites, usually referred to as EM [57,58]. The parent estrogens are irreversibly oxidized in the cytochrome P450 dependent pathway by hydroxylation at the C-2, C-4 and C-16 positions of the steroid ring forming hydroxylated metabolites. The main and mots studied metabolites include 2-hydroxyestrogen (2-OH-E), 4-hydroxyestrogen (4-OH-E) and 16-hydroxyestrogen (16α-OH-E) have significant estrogenic activity (Figure 4). Those metabolites are further transformed by conjugation with a methyl group, glucuronic acid, and sulfuric acid (forming methoxy-metabolites, glucuronates and sulfates, respectively). Thus, many authors point out the necessity of studying a wide panel of estrogens, including minor metabolites in order to fully understand their influence on human physiology as well as the etiology and progression of various pathological states [59,60]. This relatively new approach needs easily accessible and reliable bioanalytical methods to determine their concentrations in human biofluids and tissues.

**Figure 5:**
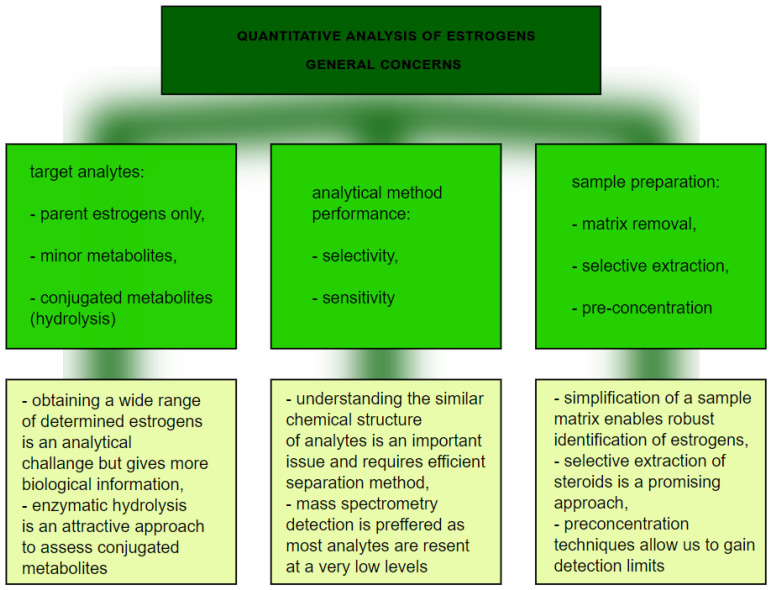
The graph presents quantitative analysis of estrogens.

From the analytical point of view, this is not a straightforward task for several main reasons (Figure 5) Firstly, the analytical technique needs to have enough selectivity to differentiate between chemically similar compounds, so an efficient separation technique is required. To achieve that, chromatography is applied, with high performance liquid chromatography coupled to mass spectrometry as a method of choice [61,62]. Practically, it is not possible to separate lipophilic parent estrogens, their hydroxylated metabolites, and much more polar conjugates with glucuronic and sulfuric acids. In order to avoid the development validation of separate LC-MS methods for polar and nonpolar analytes, enzymatic hydrolysis is typically involved as a sample preparation step. β-glucuronidase/sulfatase from Helix pomatia has been proven to sufficiently hydrolyze estrogen metabolites [62]. The involvement of enzymatic cleavage enables us to gain detailed information about a wide range of estrogen metabolites. Secondly, due to the low levels of many of the above mentioned metabolites, the high sensitivity of the analysis is a critical issue. Sensitive quantification depends on the detection method and sample preparation. Mass spectrometry, despite being expensive, can detect estrogen compounds down to the pmol/L level [59]. On the other hand, extensive clean-up of a sample with simultaneous preconcentration of analytes is beneficial to improve sensitivity and avoid interfering compounds. Improvement at the sample preparation step in estrogen analysis is thus still required. The application of novel selective materials, including sorbents processed by using 3D-printing, can be a promising approach, especially in a high throughput format [63,64]. More selective extraction utilizing specific sorbent-analyte interactions can potentially further improve quantification of a wide range of estrogens. In particular, boronate affinity solid-phase microextraction, as was previously claimed to be useful for diol-containing compounds [65], seems to be an attractive approach. 

The main role of estrogens in a woman’s body is to shape secondary and tertiary sexual characteristics, which affects the development of external genitalia, as well as the fallopian tubes, uteri, vaginas and nipples. Estrogens also perform a key function in the male body—they condition the development of the male reproductive system [55,66,67,68,69,70,71,72]. 

Estrogens also have a positive effect on the cardiovascular system, among other factors, by lowering total cholesterol [60], shaping the blood lipid profile and also affecting the musculoskeletal system by stimulating the repair process of damaged muscle fibers [56,71]. Notably, during menopause, there is a decrease in estrogen levels, which results in a decrease in muscle mass, as well as osteoporosis. The lowest biological activity is shown by estriol, which is considered to be the weakest of estrogens, being a product of estrone and estradiol metabolism. Estrone, in turn, exhibits a markedly higher biological activity [56,68,69,70,71]. Finally, the type of estrogen with the highest activity is a 17β-estradiol, with a potency of about 5 to 10 times greater than the former type. Both types of estrogen receptors—ERα and ERβ—have a relationship with the heat shock proteins (HSP) complex. In addition, estrogen receptors have the ability to form heterodimers and homodimers [47,68,69,70,71].

## 5. Estrogens in Etiopathogenesis and Therapy of Lung Cancer

It is well known that estrogens can cause carcinogenicity. The impact of estrogens is noted in female cancers, for example breast cancer, or in the case of modulation of genetic mutations. However, based on available clinical studies, it is also hypothesized that the mechanism of action of the estrogen pathway in lung cancer is similar to the one established for breast cancer [72,73,74].

Available studies indicate that estrogen affects lung carcinogenesis via non-genomic and genomic signaling. In genomic signaling, homodimers and heterodimers are formed acting as ligands, which bind to the ER nucleus. In contrast, non-genomic signaling works by means of the mitogen-activated protein kinase (MAPK1) pathways through the ER [71,75].

The endogenous metabolite of 17β-estradiol (E2) resulting from the hydroxylation and methylation of the second-position is 2-methoxyestradiol (2ME, (17beta)-2-methoxyestra-1,3,5 (10)-triene-3,17-diol). This metabolite inhibits angiogenesis by reducing endothelial cell proliferation. In addition, 2ME is an antiproliferative and anti-angiogenic agent [76,77,78,79]. 

The metabolite 2ME inhibits carcinogenic cell growth due to tubulin binding. In vitro studies show that 2-methoxyestradiol inhibits a wide range of non-cancer and cancer cell lines. It has also been shown in vitro that 2ME inhibits several stages of the andiogenic cascade, thereby inhibiting proliferation and inducing tumor cell apoptosis. Cell line growth inhibition was achieved in the lung cancer line of human origin A459 and H460 *p53* wild-type. Minor changes after treatment with 2ME occurred in H322 *p53* and H358 type *p53* cell lines. Western Blot analysis was performed, which resulted in a significant increase in *p53* protein after treatment with 2-ME. The main change observed during the study involving treatment with 2ME was an eight-fold increase in endogenous *p53* protein. The level of mutated *p53* protein remained unchanged. The *p53* protein is the major tumor suppressor responsible for regulating the cell’s life cycle and apoptosis [76,77]. 

The Charité University Clinic in Berlin conducted a study to confirm the inhibition of the growth of various cell lines with 2ME, including lung cancer. Orally administered 2-ME was combined with gene therapy and an adenovirus expressing the *p53* gene was administered intravenously. The results demonstrated that lung cancer cells that were resistant to cisplatin were particularly sensitive to 2ME [78].

Several experiments have been devoted to the metabolite 4-hydroxyestrogen (4-OH-E), which is a CYP1B1 product, and has mutagenic and carcinogenic effects [78]. Studies indicate that tobacco smoke stimulates the metabolism of 17β-estradiol to the toxic metabolite 4-OH-E. In addition, 4-OH-E levels are elevated in patients with lung cancer as compared to healthy controls. It has been hypothesized that the 4-OH-E metabolite affects oncogene mutation in the lungs and also activates ER signaling, which increases the risk of lung cancer. Last year at the Research Institute of Fox Chase Cancer Center in Philadelphia, it was discovered that the human lung can metabolize estrogen to 4-hydroxyestrogen [79]. 

Studies show differential expression of nuclear ER-β in NSCLC [80]. Nuclear expression of ER-α and ER-β was determined by immunohistochemistry. The study identified ER-β nuclear expression in NSCLC tumor tissue and control tissue correctly, in both women and men. In men, nuclear ER-β expression was found to be more frequent in adenocarcinomas of the lung [80]. 

In the case of NSCLC, research indicates stimulation of tumor growth through the expression of ER forms that interact with the epidermal growth factor receptor (EGFR) [81]. Importantly, EGFR supports the growth of NSCLC and breast cancer. The available data indicate the responsibility of HER2 and EGFR for a number of states of endocrine immunity [66]. Moreover, in response to estrogen, the ERs proliferate as a result of their interaction with ER-containing vascular endothelial cells [81]. 

Estrogens regulate the expression of miRNAs, which are found in small non-coding RNAs containing about 21–25 nucleosites [82]. The miRNA finds application in distinguishing between different subtypes of lung cancer [82,83,84]. Studies have reported that miR-124a is characteristic of NSCLC lung adenocarcinoma and miR-205 for squamous cell carcinoma, while miR-375 and miR-21-5p are highly expressed in SCLC [82,83,84,85,86,87,88]. In addition, the histological patterns of growth of lung adenocarcinomas were analyzed, showing a significant influence of miRNA expression [89]. In tumors, the presence of solid components in tumors was demonstrated when miR-212, miR-27a and miR-132 were expressed [89]. However, in order to demonstrate the possible benefits of miRNA targeted therapy in lung cancer patients, more comprehensive studies should be conducted.

Research indicates that estradiol can be synthesized locally in NSCLC, analogous to breast cancer tissue [90]. Moreover, on the basis of the obtained results, it was proved that the concentration of estradiol in the NSCLC tissues was significantly 3.7 times higher in men than in women after menopause [90]. Researchers say that the essence of this phenomenon are the circulating androgens produced by aromatase, which in the case of NSCLC and estradiol production could be the leading substrates [90]. The study determined the estradiol concentration in 59 NSCLC cases, followed by in vitro A549 NSCLC cell cultures. Forty-three of the subjects showed an increase in the concentration of estradiol in the neoplastic tissues compared to the non-neoplastic lung tissues of the patients [90]. However, in the case of in vitro studies, the increase in the proliferation of cell cultures of both A549 + ER-α and A549 + ER-β was determined. Moreover, both cell cultures were found to express aromatase. Importantly, studies show an increase in A549 cell proliferation during testosterone use. Therefore, it is suggested on the basis of the obtained studies that if estrogens, and more specifically oestradiol occurring inside cancerous tumors by aromatase, including NSCLC, and favor their development, anti-estrogen therapy would be an effective therapy in the fight against cancer [90].

Both NSCLC and breast cancer are entities that frequently occur in everyday pathological diagnosis [91]. However, it should be remembered that both disease entities in the form of lung metastatic breast cancer and primary lung cancer are treated completely differently [91]. Typical immunohistochemical markers in the differential diagnosis of breast cancer are: HER2—tyrosine kinase receptor encoded by HER2—growth-promoting protein, ER, MAMG—mammaglobion, GATA3—GATA 3 binding protein (zinc finger transcription factor) and PgR—the steroid hormone progesterone receptor [91]. However, in the case of NSCLC, the most frequently used immunohistochemical markers are: TTF-1—thyroid transcription factor 1, Napsin A, CK7—cytokeratin 7, *p63*, *p40* and CK5—cytokeratin 5 [91]. In addition, molecular tests for mutations are performed to diagnose NSCLC activators in the EGFR gene [91]. Due to the limited research on the immunohistological expression of NSCLC markers, a study was conducted using clinical variables and staining results for CK5/6, *p40*, TTF-1 and napsin A [91]. An analysis of 1291 samples of NSCLC patients with successively diagnosed adenocarcinoma (ADC) was performed—636 patient samples, squamous cell carcinoma (SqCC)—536 patient samples, large cell carcinoma (LLC)—65 patient samples, polymorphic carcinoma (PC)—34 patient samples, and large cell neuroendocrine carcinoma (LCNEC)—20 patient samples. Most of the patients had disease stages I to III [91]. In addition, 380 patients were women with more ADC than SqCC, while the remaining 911 patients were men. ER-positive tumors were found to be much more common in women than in men. On the basis of the conducted studies, the expression of all five markers was found in many patients, thus it can be concluded that the interpretation of tumor markers is important in the differential diagnosis [91].

Estrogen is known to induce ERβ-mediated cell growth in NSCLC [92]. Moreover, high levels of circulating interleukins 6 (IL6) are associated with poor prognosis for NSCLC; however, the determination of the specific role of IL6 in NSCLC is not fully understood and requires a lot of research [92]. One of the studies assessed both the biological effects as well as the expression of interleukins in NSCLC cells after treatment with 17β-estradiol (E2) [92]. The expression of IL6/ERβ in 289 NSCLC samples was determined via immunohistochemistry [92]. The study included A549 and H1793 non-small cell lung cancer cells [92]. Cells were treated with E2. Their expression levels were determined sequentially by means of ELISA, western blotting and immunofluorescence staining [92]. The study also used an animal xenograft model to determine and observe differences in IL6 and ERβ expression in NSCLC tumor growth [92]. Research showed an increased increase in both ERβ and IL6, which was closely related, the researchers indicated, to either increased metastasis or decreased differentiation [92]. Indeed, the study showed ERβ mediated regulation of IL6 expression through the PI3K/AKT and MAPK/ERK pathways due to the use of E2 [92]. Importantly, an increase in malignancy of NSCLC cells was also found due to the regulation of E2 on IL6/ERβ [92].

Experimental research indicate that the ER potentially promotes NSCLC progression via modulation of the membrane receptor signaling network, composed of the GSK3β/β-catenin, Notch1 and EGFR pathways [93]. Furthermore, one of the treatments for lung cancer may be anti-estrogen therapy. Additionally, 17-β-estradiol is produced by aromatase activity, which in turn influences the control of estrogen levels in the lung cancer microenvironment [94,95]. Clinical studies suggest that aromatase inhibitors are a good therapeutic option for lung adenocarcinoma [94,96]. Aromatase inhibitors are classified into classes I and II: (I) irreversible steroid inhibitors; (II) non-steroidal inhibitors [94,96]. Sulfotransferases activated by e.g., dexamethasone are also used in the treatment of hormone-dependent tumors [94,97]. Preclinical studies indicate inhibition of A549 cell tumor growth, while lowering estrogen levels [94,96]. Fulvestrant, an estrogen receptor degradator, is also used in NSCLC research [98]. According to the data, fulvestrant causes greater sensitization of the NSCLC tumor to chemotherapy and reduces the mesinochemical features [94,97]. Due to the fact that one of the leading elements of the patient’s immune profile are steroid hormones, next to chemotherapy, radiotherapy or surgery, immunotherapy is an effective lung cancer treatment strategy [94]. The development of personalized medicine is conditioned by numerous preclinical and clinical studies taking into account sex differences or the expression of hormonal markers, in which the response to therapy in patients with NSCLC, survival as well as pathological and clinical features are tested [94].

## 6. Current Clinical Trials Registered for Non-Small Lung Cancer and Estrogens with Completed Status with Results

Currently, as of 20 December 2020, 3 clinical trials are registered on clinicaltrials.gov for lung cancer:

1. Study Evaluating the Addition of Fulvestrant to Erlotinib in Stage IIIB/IV Non-Small Cell Lung Cancer—ClinicalTrials.gov Identifier: NCT00592007, disease entity: stage IIIb/IV NSCLC; drug treatment: fulvestrant, erlotinib; clinical trial is aimed at determining the effectiveness of the combination of fulvestrant which inhibits the access of estrogen to the tumor with erlotinib. Only patients who express estrogen are eligible for the study. Moreover, estrogen sensitivity was tested on previously removed tumor samples [99].
2. Fulvestrant and Anastrozole as Consolidation Therapy in Postmenopausal Women With Advanced Non-small Cell Lung Cancer—ClinicalTrials.gov Identifier: NCT00932152; target audience: postmenopausal women, NSCLC; Drug: fulvestrant (Faslodex), anastrozole (Arimidex), bevacizumab (Avastin), best supportive care; clinical trail included the assessment of 17β-estradiol, VEGF, E-selectin, thrombospondin-1 and IGF-1 levels and other plasma biomarkers. Evaluation of biomarkers such as ERα, ERβ, PR, VEGF and aromatase expression. Archiving of tumor tissue was also used in the study [100].
3. Alisertib in Adults With Nonhematological Malignancies, Followed by Alisertib in Lung, Breast, Head and Neck or Gastroesophageal Malignancies—ClinicalTrials.gov Identifier: NCT01045421; disease: advanced nonhematological malignancies, non-small cell lung cancer, small cell lung cancer, metastatic breast cancer, head and neck squamous cell carcinoma, gastroesophageal adenocarcinoma, drug: MLN8237 (Alisertib); in lung cancer, the chemo-sensitive, chemo-resistant population was analyzed, in breast cancer, ER2 and ER2 were analyzed. HR + = positive estrogen or progesterone receptor, both SCLC and NSCLC patients received 50 mg of MLN8237 orally twice daily for 7 days, consecutively 14 days off [101].

## 7. Conclusions

Extensive data, in vitro and in vivo studies indicate a significant role of the female sex hormone β-estradiol in the etiopathogenesis, clinical treatment and prognosis of NSCLC. This manuscript focuses on a review of the available data describing the hormonal difference between the sexes in the development of lung cancer. 

Estrogen activity in the growth of NSCLC tumors has been confirmed by a number of studies, and lowering the level of estrogen hormones could have a positive effect on antitumor activity in this area.

There have been several reports suggesting an upward trend in the incidence of lung cancer in women. Compared to men, a much more common tumor suppressor *p53* mutation was observed in women with NSCLC [22,24,61].

There is enough in vivo and in vitro evidence that female sex hormones are an important factor in the development of neoplastic tumors, which are mitogenic and carcinogenic. Research indicates that women are more predisposed and exposed to adenocarcinoma, while men are more likely to suffer from squamous cell carcinoma [90].

There are two types of estrogen receptors in lung cancer cells: ERα and ERβ. The ERα receptor has been detected in a number of lung cancer cell lines, and interestingly, the ERβ receptor is a prognostic marker in NSCLC [37,38,39,40].

The manuscript presents the molecular basis of lung cancer in women. The authors point to a number of genetic abnormalities that may be closely related to the increased incidence of lung cancer among women. Advances in medicine and molecular diagnostics create an opportunity for more effective anti-cancer therapies and detection of lung cancer at an earlier stage. Role of estrogens in pathogenesis and diagnosis of lung cancer therefore still needs to be elucidated. The review was based on extensive literature emphasizing the important role of estrogen and estrogen receptors in the progression and development of NSCLC.
